# The 2026 Men’s FIFA Football World Cup: Evidence-Based Guidelines to Protect Player Health and Performance from Environmental Challenges

**DOI:** 10.1007/s40279-026-02398-4

**Published:** 2026-03-24

**Authors:** Chris J. Esh, Sarah Carter, Valérie Bougault, Olivier Girard, Dina C. Janse van Rensburg, Bryna C. R. Chrismas, Tim Meyer, Lee Taylor

**Affiliations:** 1 Aspetar, Orthopaedic and Sports Medicine Hospital, Doha, Qatar; 2 School of Sport, Exercise and Health Sciences, National Centre for Sport and Exercise Medicine (NCSEM), Loughborough University, Epinal Way, Loughborough, LE11 3TU UK; 3 Faculty of Health, Exercise and Sports Science, Sport, Performance, and Nutrition Research Group, School of Allied Health, Human Services and Sport, La Trobe University, Melbourne, Australia; 4 LAMHESS, Université Côte d’Azur, Nice, France; 5 School of Kinesiology, University of British Columbia, Vancouver, BC Canada; 6 School of Human Sciences (Exercise and Sport Science), The University of Western Australia, Perth, WA Australia; 7 Section Sports Medicine and SEMLI, Faculty of Health Sciences, University of Pretoria, Pretoria, South Africa; 8 Medical Advisory Panel, World Netball, Manchester, UK; 9 Institute of Sport and Preventive Medicine, Saarland University, Saarbrücken, Germany; 10 School of Sport, Exercise and Rehabilitation, Faculty of Health, University of Technology Sydney (UTS), Sydney, Australia; 11 Human Performance Research Centre, University of Technology Sydney (UTS), Sydney, Australia

## Abstract

The Men’s 2026 Fédération Internationale de Football Association (FIFA) Football World Cup (FWC) will take place across the USA, Mexico and Canada, with host cities spanning ~ 4300 km east–west and ~ 4000 km north–south. The geographical distribution exposes players to environmental challenges that can negatively impact their health and performance, including: (i) extreme heat; (ii) altitude; (iii) air pollution and seasonal allergens; and (iv) travel. FWCs have never presented this combination of extreme environmental factors. Extreme heat is expected in 14 out of the 16 host cities, with historical maximum wet-bulb globe temperatures ranging from 21 to 35 °C (mean ambient temperatures: 19.1–32.7 °C). Matches in Guadalajara (1566 m) and Mexico City (2240 m) will take place at altitude. Travel requirements and increased human activities during mega-events increase the susceptibility of players/athletes to, and risk and spread of, airborne illnesses. Changes in air pollution and allergens from players’ domestic club locations and between host cities will also impact player health. Circadian misalignment (up to 19 time zones may be crossed to reach team base camps) and travel fatigue (up to 3 time zones crossed and a 7-h in-tournament flight time) may impact players’ mental and physical health, decrementing athletic performance. Teams adopting evidence-based guidelines to mitigate the impact of these environmental challenges will be best prepared to protect player health and performance during the tournament. Therefore, this review presents the predicted environmental challenges using the best available data and models and provides evidence-based long- and short-term strategies to best mitigate their impact. Relevant clinical considerations for each challenge are also outlined.

**Supplementary Information:**

The online version contains supplementary material available at 10.1007/s40279-026-02398-4.

## Key Points

**Table:** 

Typically, Fédération Internationale de Football Association (FIFA) World Cups present one or more environmental challenges to players’ health and performance, including, heat, altitude, air pollution and allergens, and travel, but never has one tournament presented such a combination of extreme environmental factors as the Men’s 2026 FIFA World Cup across the USA, Canada and Mexico (2026 FWC).
Evidence-based preparation and mitigation strategies exist for the environmental challenges that players will face at the 2026 FWC; however, these often lack compatibility with football and FWC practice owing to a lack of football-specific data.
Existing evidence-based preparation and mitigation guidelines are presented with football specificity where possible, and teams that effectively integrate these guidelines into their practice will be best equipped to protect player health and performance amid the environmental challenges expected at the 2026 FWC.

## Introduction

**Fig. 1:**
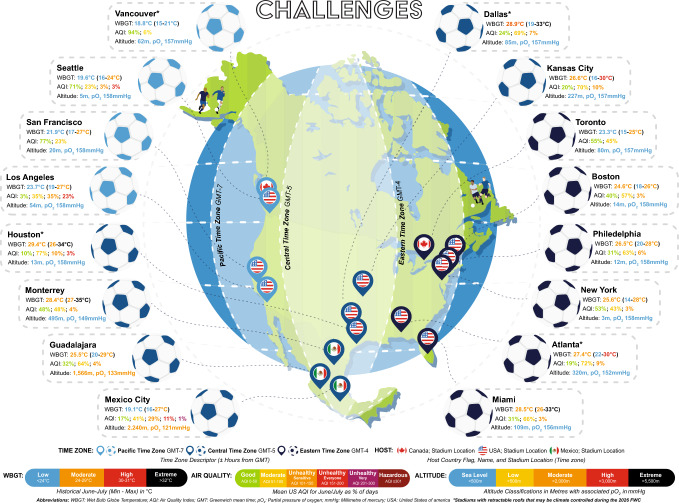
An overview of players’ environmental challenges at the Men’s 2026 FIFA Football World Cup. WBGT data were obtained from references [1, 3]. Methods used to determine air quality data are presented in the Supplementary Material. WBGT, wet-bulb globe temperature

The Men’s 2026 Fédération Internationale de Football Association (FIFA) World Cup (FWC) takes place across 16 cities within the USA, Mexico and Canada, spanning ~ 4300 km (~ 2700 miles) east to west and ~ 4000 km (2400 miles) north to south. This geographic spread will expose players to environmental challenges that may negatively impact their health and performance, including: (i) extreme heat; (ii) altitude; (iii) air pollution, seasonal allergens; and (iv) travel (Fig. 1).

Whilst the prospective risk of extreme heat at the 2026 FWC has been outlined [1–3], limited player-specific strategies have been developed/proposed to mitigate the risk of extreme heat on health and performance at the 2026 FWC. The mean historical wet-bulb globe temperature (WBGT) at the timepoint of the tournament for the 16 host cities ranges from 18.8 °C to 29.4 °C [maximums: 21–35 °C [1, 3]; ambient temperatures: 26.7 °C (19.1–32.7 °C)] [3]. These historical WBGTs represent the minimum that players/teams should expect to experience as the probability of record temperatures increases [3, 4] alongside heatwaves becoming more frequent, longer and intense [5]. Even host cities with historically ‘mild’ WBGTs (e.g. Vancouver, Seattle: 15–24 °C) could experience extreme heat, whilst already hot locations (e.g. Monterrey, Houston, Miami: 26–35 °C) may see exacerbated extreme/dangerous WBGTs (> 32 °C). In the heat, players obtain higher body tissue temperatures [core (Tc), skin (Tsk) and muscle (Tmu) temperature] than in temperate conditions [6], reducing physical [7, 8] and cognitive [9–11] performance, and increasing the risk of exertional heat illness (EHI) and/or stroke (EHS) [12]. Within football, data from professional leagues [Spanish La Liga, German Bundesliga 1/2, Australian A-league, Japanese J-League and Turkish SüperLig (peak WBGT: 25.1–29.6 °C)] [13–15] and the 2014 FWC (Brazil, peak WBGT not specified) [16] generally show a decrease in match intensity [14], including reduced capacity to perform high-volume match actions [15], reducing distances covered at a high speed [13, 16]. This often results in modified (deliberate or otherwise) team tactical strategies/playing styles [e.g. lower (or less) high-speed running, and greater (or more) lower-speed running; shorter passes but higher success rate of passing] to counter the impact of the heat [13, 16]. However, extreme heat is not the only environmental factor players will face at the 2026 FWC.

Matches in Guadalajara (1566 m) and Mexico City (2240 m) will take place at moderate altitudes. Teams based at altitude gain approximately a half-goal advantage for every 1000 m increase in altitude compared with low-altitude teams [17]. Reduced partial pressure of oxygen (*p*O_2_) at altitude decreases aerobic capacity and recovery from high-intensity work [18]. At altitude, players will also have to contend with increased neuromuscular fatigue and altered match pacing [19], likely necessitating tactical adjustments (e.g. slower game pace to reduce fatigue) [20]. Air travel [21] and major sporting events [22] per se increase risk and spread of airborne illnesses. Air pollution [e.g. ozone (O_3_) and particulate matter (PM)] and seasonal allergens (e.g. tree/grass pollen) can individually and in combination elicit, and exacerbate, unwanted illness, asthma and allergy-associated pathologies and symptomatology (Table 1) [23] that conspire to affect player health [24] and performance [25].

Air pollution and allergens are both hyper-local [26] and variable [27] in their presentation. Wildfires that have increased in frequency and severity close to several stadium locations [Los Angeles (LA), San Francisco, Seattle and Vancouver [28, 29]] intensify many of these unwanted triggers. Given the disparate multi-country nature of the host cities, mitigating these acute environmental challenges will be particularly difficult [30].

Finally, many players will arrive at the 2026 FWC via long-haul travel (e.g. crossing up to 19 time zones), which misaligns individual biological clocks, leading to sleep disturbance alongside increased susceptibility to physical and mental illness, clearly not conducive to optimal performance [21, 31–33]. During the tournament, jet lag symptoms should be limited as teams will only cross up to three time zones (GMT-4, GMT-5 and GMT-7—maximum potential in-tournament flight time of 7 h). However, players may still accumulate travel fatigue characterised by fatigue, disorientation and headaches, which can impact health and performance with similar symptomatology to jet lag [34, 35].

Global media coverage highlighted the real-world manifestation of these concerns occurred at the FIFA Club World Cup 2025 (2025 CWC), a competition largely regarded as a proxy for the 2026 FWC. Players were hospitalised for acute gastroenteritis alongside multiple adverse heat-related events (some not treated in line with consensus guidelines [12, 36, 37]) and symptomatology. Many coaches and players were vocal in the media, citing difficulties training in the heat and high levels of discomfort during matches due to extreme temperatures.

**Table 1:** An overview of the health issues and symptoms associated with the environmental challenges at the 2026 FIFA World Cup

Challenge	Issue	Symptoms
**Extreme heat**		
	Exertional heat illness	1. Dizziness 2. Headache 3. Nausea 4. Unsteady and general weakness 5. Tachycardia 6. Muscle cramps 7. Fatigue
	Exertional heat stroke	1. Any/all from exertional heat illness 2. CNS dysfunction (confusion, disorientation, personality changes, collapse/seizure) 3. High Tc (typically ≥ 40 °C)
**Altitude**		
	Acute mountain sickness	1. Poor sleep 2. Fatigue 3. Headaches 4. Nausea
**Air pollution, seasonal allergens and airborne illnesses**		
	Air pollution	1. Cough 2. Chest tightness 3. Decreased lung function
	Seasonal allergens (e.g. tree and grass pollen)	1. Nasal obstruction 2. Nasal itching 3. Sneezing 4. Rhinorrhoea (i.e. runny nose)
	Airborne illnesses (e.g. upper and lower respiratory tract infections)	1. Any/all from air pollution/seasonal allergens 2. Fever 3. Muscle aches
**Travel**		
	Jet lag	1. Sleep disturbance 2. Fatigue 3. Impaired cognitive and physical performance 4. Irritability 5. Mental health concerns 6. GI issues
	Travel fatigue	1. Fatigue 2. Disorientation 3. Headaches

*CNS* central nervous system, *Tc* core temperature, *GI* gastrointestinal

Interestingly, although one or more of the outlined environmental challenges have presented at previous FWCs (e.g. Brazil 2014—heat/travel, South Africa 2010 and Mexico 1970/1986—altitude and sea-level air pollution, etc.), never has one FWC presented such a combination of extreme environmental factors for teams to mitigate, with heat [5], pollution [38] and allergens [39] now exacerbated by climate change. To compound the complexity of the challenges facing teams at the 2026 FWC—despite available evidence-informed preparatory and mitigative strategies relative to these environmental challenges, across a myriad of sports, including elite athlete samples—football-specific studies/data are rare. This results in football-specific practice often being based on data from other sports and/or mechanistic/physiologically oriented research with limited external/ecological validity to football. Together, these challenge the creation of 2026 FWC-specific guidelines to protect player health and performance amidst the environmental challenges that will be faced.

To best describe the environmental challenges that players will face at the 2026 FWC a variety of sources and calculations have been used:

- **Heat:** the prospective heat risk has been extensively outlined using evidence-informed, consensus-driven and transparent modelling [1–3]; the data presented in the current article have therefore been drawn from these models.
- **Air pollution and allergens:** air pollution and/or air quality data were obtained by one of the authors (V.B.), the specific methods and data acquisition sources have been outlined in the supplementary material. Historical allergen data are not freely available and thus, have not been modelled within the current article.
- **Altitude:** the elevation and respective atmospheric pressure data for the host cities/stadiums in Mexico are commonly available and are not based on historical/modelled data.
- **Travel:** Fact-based calculations are required for teams to understand/determine the level of disruption long-haul travel will have on their players. As an example, New Zealand (GMT + 12) travelling to the USA, Canada or Mexico (GMT-4 to GMT-7) will cross at least 16 and up to 19 time zones, depending on their team base camp location.

**Fig. 2:**
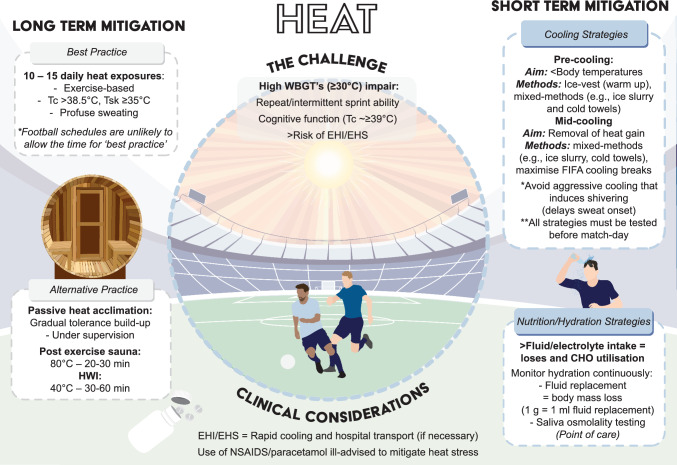
Heat at the Men’s 2026 FIFA Football World Cup. The challenge, mitigation strategies and clinical considerations. *WBGT* wet-bulb globe temperature, *Tc* core temperature, *Tsk* skin temperature, *EHI/EHS* exertional heat illness/stroke, *HWI* hot water immersion, *CHO* carbohydrate, *NSAIDs* non-steroidal anti-inflammatory drugs

**Fig. 3:**
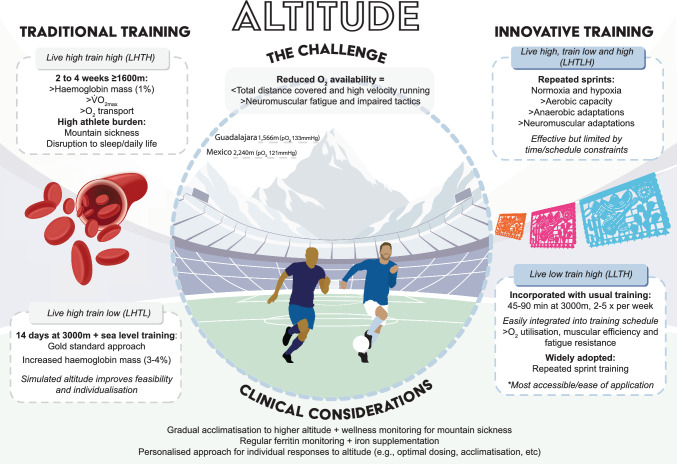
Altitude at the Men’s 2026 FIFA Football World Cup. The challenge, training strategies and clinical considerations. $\dot{V}$*O*_*2max*_ maximal oxygen uptake, *O*_*2*_ oxygen

**Fig. 4:**
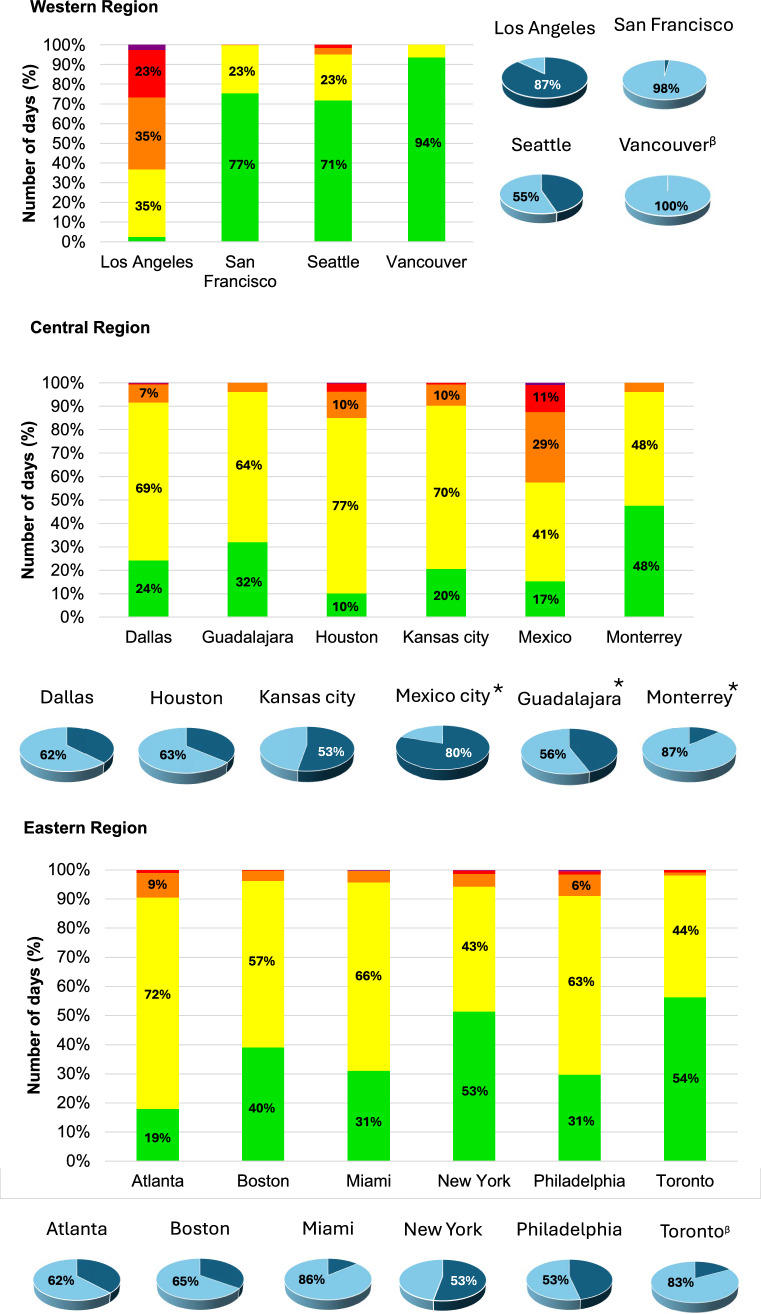
Mean number of days (%) in June and July from 2019 to 2024 in each US Air Quality Index (AQI) category in recent years. The pie chart shows the percentage of days where the main pollutant is ozone (O_3_; dark blue) or particulate matter with a size < 2.5 µm (PM 2.5; light blue), and AQI is from ‘moderate’ to ‘hazardous’. Colours: green (AQI from 0 to 50): good; yellow (AQI from 51 to 100): moderate; orange (AQI from 101 to 150): unhealthy for sensitive individuals; red (AQI from 151 to 200): unhealthy; purple (AQI from 201 to 300): very unhealthy; maroon (AQI = 301 and above): hazardous. Dark blue: O_3_; light blue: PM 2.5. For US cities, US Environmental Protection Agency (EPA) governmental data were used. ^β,*^Different data sources were used for Canadian and Mexican cities. The search methodology is described in detail in the Supplementary Material

**Fig. 5:**
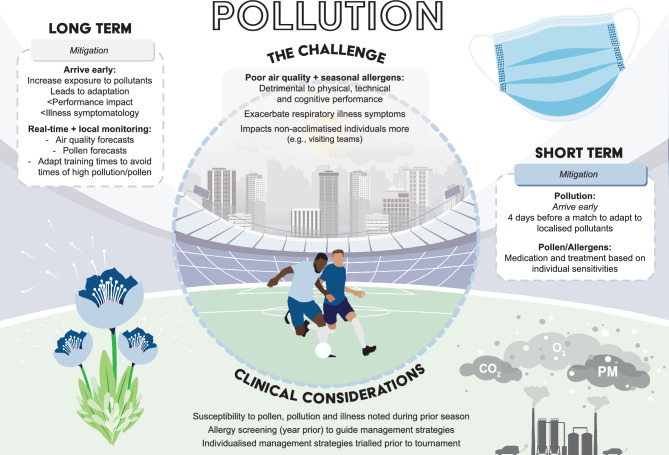
Pollution at the Men’s 2026 FIFA Football World Cup. The challenge, mitigation strategies and clinical considerations

**Fig. 6:**
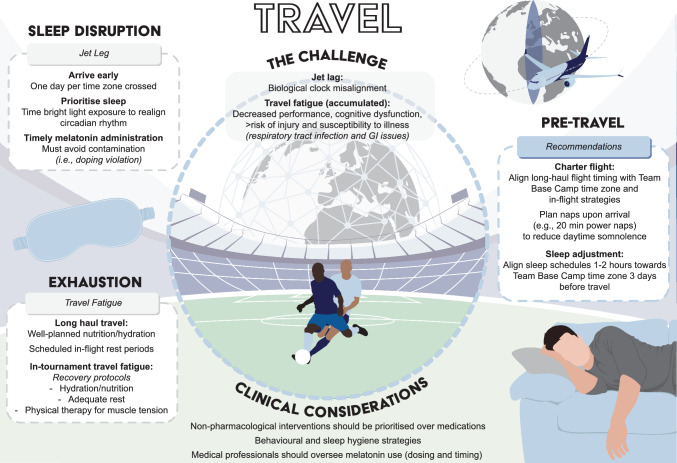
Travel considerations at the Men’s 2026 FIFA Football World Cup. The challenge, mitigation strategies and clinical considerations. *GI* gastrointestinal

**Fig. 7:**
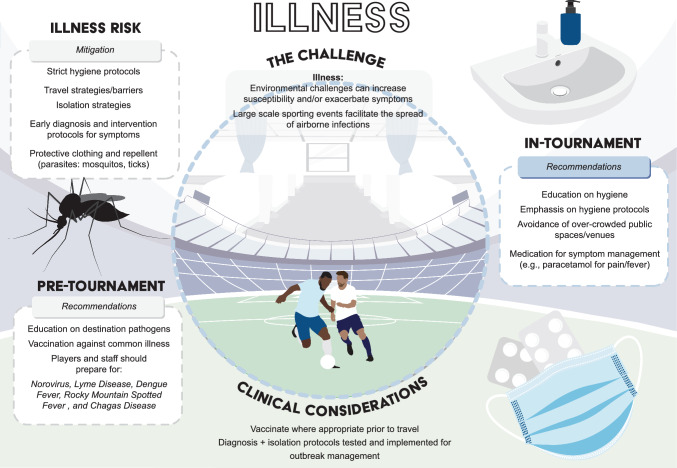
Illness at the Men’s 2026 FIFA Football World Cup

The aim of this article is to outline the current and available evidence-based guidelines that allow players/teams to prepare for and mitigate the impact of these environmental challenges. Figures 1, 2, 3, 4, 5, 6 and 7 synthesise the presented evidence and practical integration considerations from the manuscript. Whilst the authors have endeavoured to limit bias in their selection of evidence to base these practical recommendations upon, it is important for readers to consider potential bias carefully within any framework of adoption. A partner review to this article provides a framework that will help teams integrate the guidelines presented here into their 2026 FWC practice [40].

## Extreme Heat at the 2026 FIFA World Cup

### The Challenge

Football match play elicits increased body tissue temperatures that are exacerbated in hot environmental conditions [6]. In two experimental matches (professional Scandinavian players), mean peak Tc was 39.7 °C in hot (~ 43 °C) compared with 38.8 °C in temperate (~ 21 °C) conditions [6]. Whilst four youth matches in Monterrey, Mexico (2026 FWC host city) were played in hot (33 °C) and moderate (25 °C) WBGT’s, mean Tc peak (moderate: 39.1 °C; hot: 39.3 °C) was similar [41]. Attaining high Tc’s has implications for:

(i) **Health** [12]: as Tc increases, players are more susceptible to EHI/EHS (≥ 39 °C—although highly variable between individuals) [12].
(ii) **Physical performance** [7, 8]: Repeat and intermittent sprint ability (associated with game-defining moments in football) [42, 43] are impaired with elevated Tc (~ ≥ 39 °C) [7].
(iii) **Cognitive performance** [9–11]: Complex cognitive tasks, such as tracking multiple stimuli and high-level decision-making [9, 11], decrease as Tc (≥ 39 °C) and Tsk (≥ 36 °C) rise [9, 11].
(iv) **Technical/tactical performance**: Elite players pace themselves (altering technical/tactical profiles) in extreme heat, modifying match-play characteristics to maintain key performance outcomes [13–15, 44, 45].

During the 2014 FWC (Brazil), distance run at high intensity and the number of sprints were reduced in matches played in high WBGT (≥ 28 °C), yet effective playing time, total distance covered and peak running speed remained unaltered [16]. Whilst in the experimental matches outlined above, in hot (43 °C) compared with temperate (21 °C) conditions, players spent ~ 25% more time in ball possession [6] and had a higher success rate for passes [6, 16], whilst temperate conditions saw higher ball gains/losses and duels, suggesting a more transitional and ‘ball-pressing’- orientated style of play [6]. Reflecting the impact of heat on performance and health, the American College of Sports Medicine (ACSM) recommends limiting intense/prolonged activity at WBGT thresholds of 26.8 °C for non-heat-acclimatised and 30.1 °C for heat-acclimatised individuals, with cancellation of exercise at 29 °C and 32.3 °C, respectively [46]. However, FIFA’s heat policy adopts in-match 3-min hydration/cooling breaks at 30 and 75 min of match-play, if WBGT is ≥ 32 °C (match delays/postponements are at the discretion of local organisers) [47]. FIFPRO’s (professional football players trade union) stance is more conservative than FIFA, with hydration/cooling breaks recommended at 26 °C WBGT and matches delayed/postponed if WBGT ≥ 28 °C [1]. For context, even in widely regarded ‘hot’ leagues (e.g. Australian A-League – played across the Australian summer), the FIFA threshold is rarely reached, yet player health and performance are robustly challenged [14], as seen at the 2025 CWC. Within the context of the 2026 FWC, FIFA announced (7th December 2025 [48]) that all matches regardless of environmental conditions will adopt 3-minute cooling breaks. As previously mentioned, the prospective heat risk has been extensively modelled [1–3]), revealing that 14 of the 16 host cities typically experience June/July days that exceed 28 °C WBGT [3], with 6 potentially reaching maximum WBGT between 30 °C and 35 °C [1]. On the basis of maximum historical WBGT:

(i) 56% of venues will exceed FIFPRO’s recommendations (WBGT ≥ 28 °C [1]) for delay/postponement.
(ii) 25% of venues will exceed FIFA’s 32 °C WBGT threshold for cooling breaks.
(iii) 25% of venues will exceed ACSM’s cancellation limit for heat-acclimatised (32.3 °C WBGT) and 44% for non-heat-acclimatised (29 °C WBGT) individuals.

These figures could increase as heatwaves’ duration, frequency and intensity are increasing [5], and could exacerbate the prevalence and severity of heat stress players experience. Of note, 4 stadiums (Atlanta, Dallas, Houston and Vancouver) at the 2026 FWC have permanent/retractable roofs and can be climate controlled that, if employed, will reduce heat stress-based challenges during these games. Nevertheless, extreme heat stress will impact players during training/matches at the 2026 FWC. Thus, given (i) to (iii) above, adoption of evidence-based heat mitigation strategies (adapted to be practice-compatible within a football tournament context), short- and long-term in their nature, will be imperative to protect player health and performance. Whichever approach is adopted, it must in some way favourably alter the conceptual heat-balance equation [49]. Effective heat acclimation and/or acclimatisation (HA) can lower resting Tc [50] alongside maximising evaporative heat loss through optimised total body skin wettedness (e.g. maximal sweat secretion) [51]. In combination with HA protocols, cooling strategies can limit the rise in Tc during a warm-up [52] and/or reduce pre-exercise Tc [53].

### Long-Term Heat Preparation Strategies

The most effective strategy to mitigate the impact of heat stress on health and performance is active (i.e. exercise-based) HA, with several evidence-based guidelines available [49, 50, 54–59]. HA induces a range of interrelated psycho-physiological adaptations that enhance thermoregulation, reduce physiological (cardiovascular and renal) strain, lower EHI/EHS risk and improve athletic performance in the heat [60]. Key adaptations include (although are not limited to): (i) reduced resting and exercise Tc, Tsk and heart rate (HR); (ii) earlier onset and increased rate of sweating [61, 62] and skin blood flow [63]; and (iii) improved fluid balance through increased plasma volume (PV) and reduced electrolyte loss in sweat [64, 65].

The optimal HA strategy is a long-term HA (LTHA) protocol, with 10–15 consecutive days of exercise-based heat exposures (≥ 60 min per day), eliciting Tc ≥ 38.5 °C, Tsk ≥ 35 °C and profuse sweating [50, 60], which are necessary to induce significant sudomotor (e.g. improved sweating response) [50, 66] and haematological (e.g. PV increase) [50, 67] adaptations (Fig. 2). However, the 2026 FWC begins on 11 June 2026, shortly after the conclusion of domestic and continental competitions in Europe (16–24 May 2026), where > 70% of 2022 FWC players were based [68], with the Union of European Football Associations (UEFA) Champions League final scheduled for 30 May 2026. Therefore, it is unlikely that teams/players will be able and/or willing to practically integrate a 10–15-day HA protocol between the end of their domestic season and the start of the 2026 FWC. This is a common challenge within elite team sports [69, 70], given LTHA can increase: (i) internal training load [71]; (ii) the rate at which the body utilises fuel [72, 73]; and (iii) energy expenditure during exercise [74], potentially interfering with football-specific training/preparations/recovery/taper [70]. In combination with chronic overload and residual match/training fatigue (e.g. ≥ 60 matches per season [75]), the additional workload represents another logistical/practical barrier to HA implementation [76].

A short-term HA (STHA) strategy may better balance the logistical/practical challenges and considerations above, although they will still present. A 5-day heat camp with a regular training schedule in elite female Rugby Sevens players elicited some positive cardiovascular HA adaptations (e.g. lower exercising HR) with variable resting Tc responses [66]. STHA protocols of 5 days are sufficient to reduce resting/exercising Tc [77, 78] and HR [67, 79]. Although, there is no elite football-specific data, in semi-professional players those who exhibited the largest haematological adaptations from a 6-day heat-acclimatisation training camp were able to maintain their in-match running performance in the heat compared with temperate conditions [80]. Arriving early at the 2026 FWC and training in the local environment can allow players to naturally heat acclimatise [56]. For teams based in the temperate host cities (e.g. Seattle, Vancouver) this strategy may not be compatible; however, they can adopt innovative and effective HA strategies that do not require a natural hot environment [81].

An HA phenotype can be procured without a heat-specific exercise component. An effective alternative HA strategy to an exercise-based protocol is passive HA (PHA). PHA typically uses a sauna (~ 80 °C [82, 83]), hot-water immersion (HWI; ~ 40 °C [84–86]), or a heat chamber (45–50 °C [87, 88]), or a combination of these [89], to elicit adaptations. Data from endurance sport populations suggest that the most efficacious PHA strategies are post-exercise/training use of sauna or HWI for a minimum of 30 min on at least 6 consecutive days [50, 81]. PHA protocols can also be employed without pre-exercise, but may not induce the same magnitude of adaptation or require a longer duration protocol to induce the same magnitude of adaptation, compared with post-exercise passive heating [50, 81]. Although football-specific data in this space are lacking, PHA represents a practical strategy to protect athlete health and performance in the heat [50, 81] given that PHA has been shown, in recreationally active males, to elicit equal and/or a greater magnitude of thermal adaptation during short- [90] and medium-term [91] protocols compared with active HA. Adopting PHA will allow teams to preserve football-specific training quality [92], and could be integrated between the end of domestic seasons and start of 2026 FWC commitments [85, 86]. Players could begin their PHA protocols during their domestic season (reliant on effective club and federation liaison/cooperation) to procure some HA adaptations before arrival at 2026 FWC. Importantly, adoption of PHA should be supervised, ideally with Tc monitoring and access to water/fluids [50, 81], and introduced gradually to build up tolerance to the heat stress and avoid EHI/EHS symptomatology [92].

Whether SHTA [93], LTHA [55] or PHA [81] are employed, the increased physiological/psychological stress of HA can be reduced by adopting an intermittent heat stress exposure protocol (i.e. every other day), albeit increasing the total number of days required to induce full HA-relevant physiological adaptations [94]. Given that many players will be arriving with high accumulated fatigue [75], it is imperative to balance the accumulated load and player recovery ahead of the 2026 FWC matches whilst attempting to acquire HA in those who are not HA and/or top-up HA [69] in those who are partially HA already. Player load (distance covered, high-speed running, HR, etc.) [95] and subjective wellness (muscle soreness, mood, energy levels, etc.) [96] that have been consistently measured in elite football players should be carefully monitored to address this. Teams should be aware that during periods of acute match congestion (≤ 4 days between matches) self-reported subjective pre-/post-match ratings of fatigue, muscle soreness and sleep duration/quality in players at international tournaments can worsen [97]. Guidance on how to integrate (duration and timing) HA into team 2026 FWC preparations are presented in a partner review [40].

### Short-Term Heat Preparation Strategies

#### Cooling Strategies

Cooling through physical body temperature reductions (i.e. Tc and Tsk [57]) and/or perceptual changes (i.e. psycho-physiological thermal sensation/comfort, perception of physiological strain [98]) can be achieved via exposure of the body’s surface to cold fluids, air or other mediums (i.e. external cooling) or the ingestion of cold fluids (i.e. internal cooling) and can be adopted pre- and mid-match to improve performance in the heat [57, 99], through manipulation of the conceptual heat-balance equation [49, 100].

**Pre-cooling:** Aims to induce a heat sink pre-exercise by lowering Tc, increasing the time it takes for players to reach a Tc where health and performance become negatively impacted [50, 101]. Cold-water immersion (CWI) is the most effective strategy to remove heat from the body [102]; however, this can be logistically and practically challenging in a team sport/stadium environment [103] and may be reliant on the provision/availability of tournament host/local organising committee resources (i.e. temperature-controlled water baths and/or ice provision [99]; this may be the case for other cooling strategies [99]). Alternatively, ice vests effectively limit the rise in Tc during a warm-up whilst achieving an activated periphery [52, 104]. In elite male/female Rugby Sevens players, Tc was 0.7 °C (mean value) lower post-warm up with ice vest use [52, 104], whilst countermovement jump (CMJ) performance was maintained (male participants [104]). In addition, physical performance metrics (i.e. GPS-based data) were unchanged during the warm-up and players self-reported no discomfort or physical restrictions from wearing the vests [52, 104]. A mixed-methods approach [internal (ice slurry) and external (ice packs on quadriceps/hamstrings)] to pre-cooling has proven effective before simulated football performance, reducing first-half Tc, Tsk and thermal sensation and increasing first-half total distance and high-speed distance [105]. Care must be taken not to induce shivering and/or impair warm-up effects. Over-cooling of Tmu may compromise early match high-speed running capacity [57, 103]. Aggressive internal cooling may also delay the onset of sweating, reducing the body’s ability to dissipate heat (i.e. sweat evaporation) [106, 107] and alter the perception of heat stress (decrease/improve). Aggressive perceptually orientated cooling (and other cooling interventions) may allow players to over-exert themselves in the early part of the game (e.g. non-optimal pacing). Although perceptual cooling has limited effects on body temperatures, improvements in thermal comfort are cited to match the impact of physical reductions in body temperatures on physical performance by reducing the perception of physiological/thermal strain [98, 99]. This, in-turn, can increase the tolerance of high Tc/Tsk, allowing for elevated intensity of physical activity inducing a greater rate of heat gain [98, 99] and negating any benefits of the employed cooling strategies that have reduced body temperatures [99] (the majority of perceptually orientated cooling data are from endurance exercise paradigms, elite or otherwise [92, 108–110]).

**Mid-cooling:** Attempts to rapidly remove heat gained in-match (i.e. reduce Tc/Tsk) and improve perceptual responses to heat (e.g. thermal sensation/comfort) [99], with CWI being the most powerful tool to reduce Tc [102], yet near impossible to implement during a match. Half-time, ad hoc breaks in play and FIFA cooling breaks all provide an opportunity for potential non-CWI mid-cooling interventions, yet many logistical challenges to adoption present [103]. At half-time, practitioners have as little as 3 min outside of player downtime and coach-led tactical instruction/interaction to employ any cooling strategies [111, 112]. This has, in part, led to conjecture regarding increasing the half-time break [113, 114]. Entering a cool/cold changing room where players can ingest cool/cold fluids (internal cooling) and utilise ice packs/ice cold towels (external cooling) that cover a large surface area of the body will enable the greatest practice-compatible strategy for removal of heat from the body [53]. FIFA cooling breaks (mandatory for all matches at the 2026 FWC: 3 min at ~22 and ~67 min of match-play) are typically implemented at 32°C WBGT which have proven effective to reduce thermal strain in laboratory-based simulated (albeit protocols had poor ecological validity and no radiant heat-load) football match play [113, 114]. A recent study in youth players observed that the 2nd half cooling breaks [cold drinks (5 °C) and towels (5–7 °C)] lowered Tc by 0.23 °C more and improved running performance (greater moderate- and high-speed running distance) compared with drinking breaks alone, resulting in a 0.32 °C lower full-time Tc [41]. FIFA policy dictates that ice-water soaked towels, cold water and cool boxes must be supplied to teams and officials [113], it is important that these obligations are fulfilled and prioritised to aid successful implementation of many of the cooling-focused mitigation measures outlined above. During these cooling breaks, providing personal shade to players would also seem sensible. Players can also use ad hoc breaks in play to engage with any viable practitioner-facilitated cooling strategies (e.g. ice towels and slurries [53], menthol mouth rinse [115]) to reduce body tissue temperatures and improve thermal comfort/perception. There have been no adverse effects of perceptually focused cooling reported in a recent meta-analysis [99], albeit the data had a strong endurance sport focus.

#### Nutrition/Hydration Strategies

Alongside increases in physiological stress, exercise in the heat also increases fluid and electrolyte losses [55] and carbohydrate utilisation [116], which should be accounted for during training and matches. In HA players that have achieved maximal sweat secretion, hot/humid environments may lead to excess sweating where limited/no evaporative heat loss occurs, increasing discomfort and the risk of dehydration [50]. Dehydration is a major factor for developing EHI/EHS [117], increasing cardiovascular and thermoregulatory strain and negatively impacting the physical/cognitive capacity of players [118]. Hydration strategies should begin upon arrival at the team base camp (i.e. the main location where a team will stay/train during the 2026 FWC) and be maintained throughout the tournament. On match days, hydration strategies should begin in the hours before match start time to ensure the absorption of fluids and allow urination to return to normal [118, 119]. Supplementing fluids with carbohydrate and electrolyte solutions effectively compensates for increased fluid/electrolyte loss and carbohydrate utilisation [120]. A range of hydration monitoring methods and how to integrate them into practice are provided in a partner review [40].

These strategies should be individualised and well-practised within training to ensure tolerability and avoid adverse effects [57]. Whilst hydration, sweating and exercise in the heat often raise concerns regarding hyponatraemia [92, 118, 121], to the authors knowledge, there are no documented cases in football. Although maintenance of body electrolyte stores alongside euhydration remain prudent across the tournament and particularly during any arduous HA interventions. Ice slurry ingestion, as well as very cold drinks and carbohydrate supplementation of fluids, can cause severe gastrointestinal distress, and tolerance must be determined before match-day use [120, 122]. Whilst cooling and hydration strategies are effective standalone strategies to alleviate heat strain, they should always be complementary to, and not used instead of, HA [57].

### Clinical Considerations

Training/competing in extreme heat increase the risk of players suffering from EHI. EHI is a syndrome associated with a raised Tc and disordered thermoregulation, which occurs on a spectrum of severity, ranging from mild to life threatening [123]. To diagnose EHI, it is imperative to obtain an accurate Tc measurement given peripheral temperature assessments lack the necessary accuracy for clinical use during EHI [124]. Symptoms of mild EHI are broad and non-specific, ranging from mild dizziness and headaches to excessive fatigue. Mild EHI should be recognised and treated urgently given the potential for progression to more severe illness, and symptoms should resolve rapidly upon cessation of exercise, entering a cool space and ingesting fluids [12]. Persistent symptoms should warrant further prompt consideration of an alternative diagnoses or EHI-associated organ damage [12]. Players who have previously experienced mild EHI may be at increased risk of future EHI [125].

EHS is the severe form of EHI and is characterised by high Tc (typically > 40 °C) with the presence of central nervous system dysfunction [126]. EHS is a time-critical medical emergency, with duration of hyperthermia associated with morbidity and mortality [127]. A treatment priority for EHS is rapid cooling, and this should be delivered in parallel with other resuscitative interventions at the scene prior to transfer to hospital [12, 117, 126]. The gold standard cooling modality is CWI [59, 128].

Ingestion of paracetamol or non-steroidal anti-inflammatory drugs (NSAIDS) has received attention for preventing EHI/EHS and/or alleviating thermal strain [129]. However, limited evidence in elite athletes supports their use to limit the rise in Tc during exercise [130], and no evidence suggests they increase Tc cooling rates or reduce the risk of developing EHI. Indeed, they may increase the risk of EHI-associated organ injury [126, 131] and other adverse side-effects due to interactions with other forms of medications and supplements that players may be taking with or without the team physicians’ knowledge [129].

## Altitude at the 2026 FIFA World Cup

### The Challenge

The 2026 FWC will feature matches (*N* = 9, Group A: 4, Group K: 2, Group H: 1, round of 32: 1, round of 16: 1) at moderate altitudes in Mexico [Guadalajara: 1566 m (~ 133 mmHg—atmospheric pO_2_); Mexico City: 2240 m (~ 121 mmHg)]. Reduced pO_2_ at these elevations impairs aerobic capacity and delays recovery during high-intensity efforts [18–20]. Maximal O_2_ consumption ($\dot{V}$O_2max_) decreases by 7–8% per 1000 m above 1500 m, impacting locomotor patterns [18]. Indeed, during the 2010 FWC (South Africa), playing above 1200 m deteriorated running performance but not technical skills [132], with a 3–9% reduction in total distance covered and up to 21% decrease in high-velocity running. Midfielders were particularly affected, showing declines in total distance covered, high-speed efforts and deceleration capacity [132]. These challenges are compounded by neuromuscular fatigue, altered pacing and impaired tactics [19]. Conversely, reduced air density at altitude may enhance sprint performance and alter ball aerodynamics [133]. Historical FIFA data from 1460 matches across ten countries in South America over a century reveal that high-altitude teams score more and concede fewer goals than low-altitude teams, with every 1000 m altitude difference giving the home team an ~ half-goal advantage [17]. Furthermore, teams at the 2010 FWC whose team base camp was at 950–1700 m doubled their chances of winning in matches played against sea-level-based teams at altitudes between 1170 and 1390 m [134]. Altitude-based teams also scored more second-half goals in the highest altitude stadiums [134]. Tailored training plans and position-specific strategies are essential to mitigate these challenges and sustain performance during altitude matches.

### Traditional Altitude Training Preparation Strategies

Traditional altitude training methods include ‘live high-train high’ (LHTH) and ‘live high-train low’ (LHTL). LHTH involves living and training at altitude (1600–2500 m) for 2–4 weeks, promoting physiological adaptations [increased haemoglobin mass (HBmass) and $\dot{V}$O_2max_] [135]. These haematological improvements enhance O_2_ transport and aerobic capacity, making LHTH effective for acclimatisation before altitude competitions. However, its practicality is limited by the abovementioned time constraints, particularly for teams preparing for the 2026 FWC [133]. Alternatively, LHTL offers a more flexible and widely regarded ‘gold standard’ altitude preparation strategy [136]. By combining altitude residence with high-intensity training at lower elevations, LHTL leverages erythropoietic benefits of hypoxia whilst maintaining training intensity [136] (Fig. 3). Research shows that 100 h of LHTH (> 2100 m) can increase HBmass by ~ 1% [137], whilst 10–14 days of LHTL can enhance HBmass by 3–4%, even in players with high baseline values, significantly improving football-specific performance [138, 139]. The use of simulated hypoxic environments (i.e. altitude hotels and normobaric hypoxic chambers) enhances the feasibility of LHTL by allowing individualised hypoxic dosing and integration into regular football training programs [140]. Altitude training camps are typically implemented to improve endurance exercise capacity/performance [141, 142], only one of many performance-defining factors in football.

### Innovative Altitude Training Preparation Strategies

The ‘live low-train high’ (LLTH) method is a practical, cost-effective altitude-training strategy, with better practice compatibility (compared with LHTH/LHTL) for players preparing for the 2026 FWC. Allowing players to live near sea level (particularly if an altitude hotel model is adopted) and preserving their routines and sleep quality whilst incorporating targeted hypoxic training sessions (which can be in an altitude chamber if technical/tactical training is not required) typically lasting 45–90 min at moderate altitude (~ 3000 m), two to five times per week [143]. LLTH minimises disruptions to training (physical, technical and tactical) whilst promoting key peripheral adaptations, such as improved O_2_ utilisation, buffer capacity and muscular efficiency [140]. Various LLTH methods exist, including systemic approaches such as continuous low-intensity training, interval hypoxic training, repeated-sprint training, sprint interval training, resistance training and local hypoxia stimuli, such as blood-flow-restricted exercise [143].

Perhaps the most widely adopted and effective strategy is repeated-sprint training in hypoxia (RSH), which improves neuromuscular efficiency and fatigue resistance through maximal short-duration efforts in a hypoxic environment [144, 145]. In highly trained youth football players, ten RSH sessions over 5 weeks were more effective than normoxic repeated-sprint training at improving agility—particularly direction changes—though they offered no additional benefits for explosive power, maximal sprinting or repeated-sprint ability (RSA) [146]. Whilst RSA per se may not be crucial to footballers’ performance, the ability to repeat/sustain short accelerations at sub-sprint speeds are likely an important factor to match performance [147]; thus, RSH is likely an effective strategy to mitigate some of the impact of altitude on performance. The ‘live high-train low and high’ (LHTL + H) method, which combines the aerobic benefits (see Sect. 3.2) of LHTL with the anaerobic and neuromuscular adaptations of LLTH, has gained popularity for significantly enhancing sea-level RSA [138]. These performance enhancements persist for up to 3 weeks post-intervention, making LHTL + H altitude training an effective strategy for pre-tournament preparation; however, in the context of the 2026 FWC, time constraints likely prohibit its use. Finally, carefully managed combinations of hypoxic and heat exposure may further amplify these benefits, although this evidence is limited to endurance athletes and strength training, and there is an absence of football-specific evidence [148]. Further guidance on the implementation of altitude-based preparation and mitigation and the potential for HA to benefit teams at altitude is presented in a partner review [40].

Altitude training (traditional or innovative methods) also requires extensive planning from experienced practitioners to avoid the additional stress of altitude increasing the risk of injury/illness and eliciting underperformance in players [141]. Teams not playing matches at altitude should consider any altitude-based training carefully. Indeed, as alluded to in Sect. 2.2, player load and wellness could be monitored to assess the extent of any additional physiological strain on players due to altitude stress.

### Benefits for Sea-Level Performance

Altitude training offers transferable benefits for sea-level performance and enhanced training responsiveness [149]. Increased HBmass and $\dot{V}$O_2max_ from altitude exposure improve O_2_ transport and aerobic capacity [150], and performance benefits are shown to be maintained for 4 weeks post-altitude camp in elite team-sport athletes [138, 151]. Shock RSH micro-cycles [145] may induce positive physiological [152] and neuromuscular [149] adaptations that benefit physical fitness [149] and sea-level performance [152]. Benefits that are complemented by psychological resilience built in challenging environments, better preparing players for the demands of near seal-level international competition [151]. The absence of football-specific data in this space however cannot be completely overcome by sound physiological reasoning alone. Balancing challenges (e.g. time constraints, increased illness risk) and football-specific theoretical advantages is a must for practitioners who consider the application of altitude training for the FWC 2026.

### Clinical Considerations

Altitude training and competition demand meticulous clinical management to mitigate adverse health effects (e.g. excessive fatigue, injury, illness [141]). Although typically experienced at altitudes ≥ 2400 m [153], unacclimatised players and/or those that fly directly to the host cities at altitude [154] could suffer from acute mountain sickness, characterised by poor sleep, fatigue, headaches and nausea [141, 155]. Gradual acclimatisation, proper hydration and regular wellness monitoring are essential to reduce these risks [155]. Training loads must be adjusted to prevent overload/overreaching whilst ensuring players’ overall wellbeing is maintained. Altitude exposure triggers erythropoiesis, increasing iron demands, making 4–6-week pre-camp ferritin assessments and individualised iron supplementation important, to support haemoglobin adaptation [142]. Current recommendations [142] are that supplementation should begin 2 weeks prior to and continue during and for days/weeks post-altitude camp and be individualised to ferritin levels [142].

Regular O_2_ saturation, HR and hydration status assessments are essential for tracking adaptation and managing fatigue during altitude exposure [141]. The timing of altitude training relative to the training or competition phase is crucial for maximising football-specific performance outcomes. Effectively managing the added stress of hypoxia as a stressor remains challenging owing to the narrow window for ‘optimal dose’ of altitude to be used [156]. Physiological responses to altitude training exhibit individual variability, and the mechanisms differentiating *fast* or *high* responders from *slow* or *low* responders remain unclear, necessitating a personalised approach for each player, aided by the knowledge of experienced altitude-focused practitioners, ideally with player familiarity [157]. Finally, teams may face both hypoxic and heat stress at the 2026 FWC. Hypoxia/heat exposure induce potentially conflicting physiological adaptations [158]. Systemic physiological adaptation to hypoxia drives haemoconcentration [158, 159] and decreases PV, whilst heat adaptations induce haemodilution and PV increases [158, 160]; therefore, it will be necessary for teams to balance their preparations. To date, concurrent heat/hypoxia acclimation protocols have produced mixed results with limited performance benefits compared with one stress alone [161–164], and physiological acclimation adaptations [161–164] appear to be evoked under a ‘worst-strain-takes-precedence’ principle [165].

## Air Pollution and Seasonal Allergens at the 2026 FIFA World Cup

### The Challenge

The 16 host cities at the 2026 FWC will expose teams to a range of air pollutants and seasonal allergens. Widespread urban and rural human activities contribute to air pollution and mega-events such as the 2026 FWC amplify human activities, exacerbating environmental impacts [166], including measured and perceived air quality [167, 168]. Given that the 2026 FWC is a summer tournament, high levels of O_3_ are expected owing to photochemical reactions between nitrogen oxides, volatile organic compounds and sunlight. Particulate matter (PM) size < 2.5 µm (PM 2.5) may remain the predominant pollutant in some cities. Detailed air quality data are provided in Table 2; Figs. 1 and 4; and Supplementary Material (for air quality data acquisition methodology, see Supplementary Material). Thus, teams should pay attention to the proximity of their training/match locations to high volumes of traffic, industrial areas and airports, which may deteriorate the air quality. Western USA/Canada [Los Angeles (LA), San Francisco, Seattle and Vancouver] have experienced unprecedented risk of wildfires [169, 170], resulting in a severe deterioration in current [169] and forecasted [28] air quality, concerning given the associated smoke and its composition can negatively impact player health and performance [171]. Several major wildfires in the Americas have led to the cancellation, postponement or disruption of major sporting events [172], with the 2026 FWC cities of LA, San Francisco, Seattle and Vancouver identified as particularly high risk [28, 29]. In the case of a large wildfire(s), all host cities at the 2026 FWC could see air quality impacted, with ~ 54% of smoke across the USA originating from western USA [169].

**Table 2:**
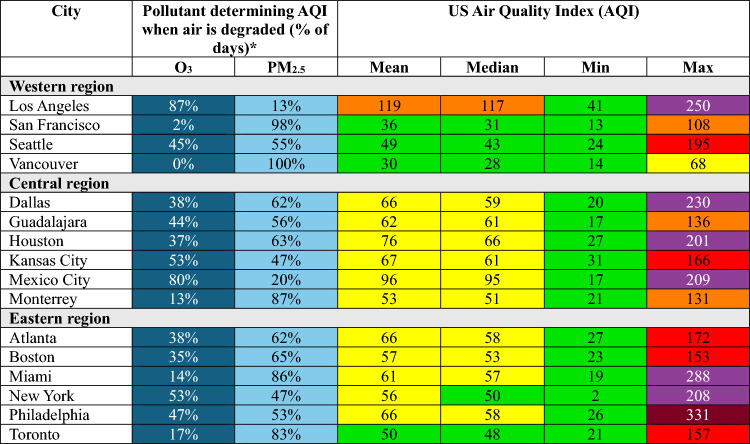
Summary of Air Quality Index per city during June and July (2019–2024)

*Pollutant determining AQI when air is degraded (AQI from yellow to maroon) is graphically presented in Fig. 4. Data acquisition and methodology is outlined in the supplementary material

*AQI* air quality index, *O*_*3*_ Ozone, *PM*_*2.5*_ particulate matter with a size < 2.5 µm

Poor air quality can pose health risks to players, with potentially severe consequences. The interaction between pollen and air pollution (plus extreme heat) could further exacerbate this situation, with grass pollen commonly present in the air of some host cities during the summer [30, 173, 174]. Historical pollen counts are not freely available and thus cannot be predicted. Teams should access reliable localised sources to assess the risk that pollen poses to sensitive players.

#### Potential Impact on Performance

Airborne pollen can negatively affect the training capacity and performance of athletes with allergies and may be exacerbated by co-occurring viral contamination and/or respiratory illnesses (illnesses are specifically addressed in Sect. 6) [175, 176]. The interaction between pollutants, particularly O_3_, and grass/tree pollen can exacerbate allergic diseases [177], although the effects of air pollution on exercise performance remain controversial. Effects vary depending on the concentration and type of pollutant, individual susceptibility and weather conditions [26]. Recent systematic reviews and meta-analyses confirm a significant effect of O_3_ on respiratory symptoms, lung function and/or physical and football-specific performance [178, 179]. Air pollution can be detrimental to physical and technical performance even at moderate [51–100 air quality index (AQI)] levels in elite football players [180, 181]. Although, the quality of research in this space is variable, there are some data from elite football to suggest that deteriorating air quality can impair performance, impairments that cannot be overcome by high skill levels of elite players [182]. Increased PM_10_ and O_3_ were associated with reduced performance during physical (30 m sprint times, change of direction score) and technical (Footbonaut football-specific assessment tool) tests, whilst increased NO_2_ impaired cognitive performance on player executive functions [180]. Other studies, assessing in-match characteristics in high-pollutant environments have observed reduced total distance covered, fewer high-intensity runs, slower sprints and changes of direction, and reduced speed and accuracy in technical tasks, including fewer passes per game, irrespective of the pollutant [25, 182–184] (Fig. 5). Visiting teams may be at a greater disadvantage than home teams that are better adapted to local air quality [182], particularly if the air quality at the tournament venue is poor and the visitors come from areas with low pollution levels.

### Long-Term Mitigation Strategies

Public health and elite athlete guidelines present several strategies to mitigate the impact of air pollution and allergies on exercise performance. To optimise training and reduce the number of days with reduced training capacity, it is essential to minimise exposure to air pollution and pollen whilst maintaining hygiene measures. However, many of the frequently proposed strategies (e.g. protective eyeglasses, facemasks, training inside [185, 186]) are not compatible with the 2026 FWC. Chronic exposure to pollution leads to some degree of adaptation, with local athletes experiencing fewer symptoms than visitors [187], and performance of adapted athletes also appears to be less affected than non-adapted athletes [182]. Sustainable mobility should be encouraged [188], and real-time and hyper-local air quality data should be monitored [189]. Given that pollutants can aggravate allergic reactions, pollen forecasts should also be monitored to ensure players adopt symptomatology-management strategies [176]. In severe cases of poor air quality and/or high pollen, exercise schedules and locations could be adjusted [189], if possible. For allergen-susceptible athletes, identifying the triggering pollen and following evidence-based management strategies, including allergen immunotherapy, can improve long-term symptom control [27, 176, 190, 191].

### Short-Term Mitigation Strategies

In preparation for the 2026 FWC, short-term mitigation strategies must address air pollutants and pollen. Adaptation to pollutants such as O_3_ typically takes about 4 days, with the first and second days of exposure often posing the greatest challenges to lung function and symptoms, particularly in sensitive individuals [192–194]. When exposure ceases, adaptations are lost within ~ 7–14 days [192]. Short-term adaptation induced by pre-exposure to low concentrations of pollutants in an environmental chamber remains uncertain [195]. Therefore, elite athlete [176] and public [192–194] health evidence dictates two key solutions to mitigate acute effects of air pollutants and pollen/allergens:

(i) Pollution: arriving at game sites at least 4 days in advance may help mitigate the acute effects of pollution [192–194].
(ii) Pollen/allergens: Personalised medication and treatment based on individual sensitivities should be implemented [176].

### Clinical Considerations

Medical management of players preparing for the 2026 FWC must pre-emptively address the effects of air pollution, pollen exposure and player susceptibility to airborne infections. With high levels of O_3_ and PM expected, combined with potential pollution spikes from wildfires, proactive health monitoring (e.g. symptomatology assessments) during the tournament will be essential. Ideally, air quality, including pollen, should have been/be monitored regularly during training and various football tournaments during the 2024/2025 seasons alongside players’ susceptibility to pollen and pollution. Screening for allergies is also recommended during 2024/2025 [196, 197] to guide further individualised management strategies.

## Travel at the 2026 FIFA World Cup

### The Challenge

Uniquely, the 2026 FWC will be hosted across three countries and 16 cities. Host nations may face limited travel-related issues; however, teams from other regions may encounter travel challenges that impact their health and performance as they adapt to varying time zones, climates and travel schedules. Although travel fatigue may resolve after 1 night of appropriate sleep, jet lag may persist over several days, especially for players crossing ≥ 10 time zones. Approaches to best protect players’ health and performance during travel are paramount. Teams that adapt swiftly and prioritise recovery may gain a competitive advantage throughout the 39-day tournament. Travel-specific challenges (Fig. 6) that teams must prepare for are:

#### Jetlag

Jetlag occurs when an individual’s biological clock mismatches with the environment due to crossing three or more time zones. This misalignment may lead to sleep disturbances, fatigue, impaired cognitive and physical performance, irritability, mental health concerns and gastrointestinal issues, ultimately affecting on-field performance [21, 31–33].

**Travel to the 2026 FWC:** Jetlag is a significant concern for players travelling from Europe (> 70% of players at the 2022 FWC played in European leagues), Asia or Africa to North America. The severity and duration of jet lag symptoms vary on the basis of: (1) the direction of travel; and (2) the number of time zones crossed. Eastward travel (i.e. advancing the body clock) results in more severe jet lag than westward travel [198].

**Group and knockout stages:** teams will not cross more than three time zones during the tournament. Even if a match occurs in LA (west coast) and the next match is in Boston (east coast), only three time zones will be crossed. However, observational studies indicate that teams travelling across any number of time zones may face more game losses [33]. Further, travel direction may affect players’ performance and game results (more profound on eastward than westward travel) [199, 200].

#### Travel Fatigue

Travel fatigue’s immediate symptoms, primarily resulting from sleep loss, dehydration and discomfort during flights, include fatigue, disorientation and headaches [201, 202]. Whilst travel fatigue is typically temporary and can resolve after 1 night of restorative sleep, frequent travel, such as during the 2026 FWC, can compound its effects, negatively impacting performance and overall wellbeing. Managing travel fatigue will be critical to maintaining peak performance, as teams will frequently travel between venues during the tournament [21] where adequate recovery time may be limited.

#### Accumulated Travel Fatigue

The cumulative burden of multiple flights may result in poorer performance, increased risk of injury, cognitive dysfunction and susceptibility to illness [203]. Across the National Basketball Association (NBA), teams typically suffer a performance disadvantage associated with high travel demands across the USA, alongside congested fixture schedules akin to what teams may experience at the 2026 FWC. Specifically, circadian misalignment, longer travel distances and frequent travel (i.e. accumulated travel fatigue) [203]. Therefore, teams must develop strategies to address this throughout the tournament [34, 35].

#### Illness

Travel, particularly long-haul (> 6 h [204]) travel, can increase susceptibility to illness, with respiratory tract infections [21] and gastrointestinal tract issues (e.g. traveller’s diarrhoea) [205] being major concerns. Illness prevention/mitigation strategies are similar during or outside of travel and are specifically addressed in Sect. 6.0 to avoid repetition of guidance between sections.

### Long- and Short-Term Preparation Strategies

To mitigate the impact of travel on player health and performance, there is considerable crossover amongst long- and short-term preparation strategies. Thus, travel preparation strategies will be presented in a single section to avoid repetition. The words ‘LONG’ or ‘SHORT’ will be used to designate strategies specific to either long- or short-term preparation. Specific guidance on how to best integrate these strategies into team travel and post-2026 FWC arrival schedules are outlined in a partner review [40].

#### Managing Jetlag and Travel Fatigue

**Long-haul travel:** For many teams, long-haul travel is necessary to reach the 2026 FWC, resulting in acute travel fatigue. Teams should implement general behavioural strategies (strategic napping, eye masks, ear plugs, optimal nutrition, caffeine/alcohol avoidance, frequent movement) for long-haul travel, including hydration protocols, nutrition planning and scheduled rest periods during flights. Compression socks have shown efficacy in mitigating the stress of long-haul travel, reducing lower limb swelling and subjective wellbeing in elite female volleyballers [206]. These measures may minimise travel fatigue and ensure that players remain physically and mentally prepared for competition [21].

**Arriving early (LONG):** One strategy for mitigating jet lag is for teams to arrive at their destination well before their matches. Research suggests 1 day of adjustment per time zone crossed when travelling east, and half a day when travelling west [34, 35], enabling players’ circadian rhythms to align with their new environment. However, this approach may face logistical challenges given the short window between the end of domestic/continental competition (late May) and the tournament start (11 June). Many teams will likely charter flights to the 2026 FWC and could/should, where possible, align their flight time with their in-flight long-haul travel strategies and team base camp time zone. They may also gradually adjust their sleep schedules 1–2 h towards the destination time zone at least 3 days before travel and use strategic napping (e.g. 20-min power naps) to reduce daytime somnolence [207, 208]. Specific jet lag management strategies may not be necessary during the 2026 FWC, as teams will not cross more than three time zones.

**Importance of sleep/rest:** Prioritising adequate rest helps players recover from travel fatigue and reduces the risk of illness [34, 35, 202]. Athletes typically experience reduced sleep during travel but can recover sleep efficiency within days of arrival [209]. Teams could consider scheduling light training sessions upon arrival to help players acclimate and promote improved sleep patterns. A post-travel rest/activity schedule should be pre-planned and outlined to players before departure (LONG). To mitigate accumulated travel fatigue, teams should schedule adequate rest periods between matches and training sessions to support physical and mental recovery, which is essential for sustaining performance levels throughout the tournament [208] (SHORT).

**Interventions to improve sleep (LONG):** Behavioural approaches (light exposure/avoidance and strategic exercise) are recommended over medication or supplements [210]. Timed exposure to bright light can help realign the circadian rhythm [198, 201]. Melatonin supplementation has shown efficacy in regulating circadian rhythms and alleviating jet lag symptoms [33, 211]. Melatonin administration can help adjust sleep patterns, particularly for travellers facing multiple time zone crossings [32, 201]. Bi-phasic melatonin formulations have greater efficacy [212, 213] and should be sourced via a prescription for a pharmaceutically available product, given concerns with contamination and inaccurate ingredient content [214]. In addition, given that evening/night matches can impair sleep and recovery [215], good sleep hygiene (SHORT) remains vital throughout the tournament [216].

**Nutrition/hydration/physical therapy:** teams should ensure that players are provided with balanced meals that support recovery and energy replenishment. The timing and composition of meals may influence the severity of travel fatigue symptoms. Implementing adequate hydration protocols before, during and after travel can help mitigate dehydration that may exacerbate travel fatigue and negatively impact performance [217, 218]. Finally, teams can utilise physiotherapy techniques post-travel to alleviate muscle tension and promote recovery [34, 35, 207].

### Clinical Considerations

Behavioural strategies and non-pharmacological interventions should be emphasised over medications to avoid detrimental effects from travel. Teams should avoid sleep medication and prioritise sleep preservation by promoting good sleep hygiene. Behavioural strategies such as strategic sleep, timed exposure to bright light, alcohol/caffeine avoidance and optimal meal timing can help players align their circadian rhythms effectively [21, 207]. Judiciously timed exercise can help resynchronise the circadian rhythm after long-haul travel, with outdoor training sessions being beneficial for mitigating jet lag symptoms. Whilst melatonin may help with jet lag recovery, players should seek medical advice before use owing to potential individual responses, side effects and anti-doping concerns. It is essential to consider dosage and timing when using melatonin [21, 207].

## Illness at the 2026 FIFA World Cup

Illnesses are somewhat beyond the scope of this article. However, environmental challenges can increase susceptibility to and/or exacerbate illness and vice versa in players at the 2026 FWC and thus, are presented briefly (Fig. 7).

### The Challenge

Large-scale sports gatherings facilitate the spread of airborne infections amongst visitors, athletes and the local population [22, 219, 220]. Infections, mainly affecting the upper respiratory tract, impact 12–16% of players [221], and respiratory tract infections represent > 57% of all reported illnesses during high-profile sporting events [222–226]. Symptoms can cause fatigue and disrupt sleep and quality of life and thus, require appropriate management [27, 175]. Lower respiratory tract infections pose the greatest risk to time-loss from training/competition; however, they are rare [222]. Travel demands on players and exposure to new environments and pathogens can also promote the spread of respiratory tract infections [21]. Gastrointestinal illnesses are common in elite athletes (all sports) travelling to different regions owing to changes in diet, water quality, hygiene and exposure to new pathogens. Traveller’s diarrhoea is a frequent concern. Pathogens, including bacterial, viral or parasitic infections [205], significantly contribute to time-loss from training/competition [227]. Furthermore, episodes of diarrhoea close to training/competition are detrimental to player health and performance [109] and, in hot environments, exacerbate dehydration and increase EHI/EHS risk [228]. Endemic and tropical diseases will also be a factor at the 2026 FWC. Examples being norovirus, Lyme disease, dengue fever and Rocky Mountain spotted fever [229, 230]. Those in Mexico (especially rural areas) should also be aware of Chagas disease [231] and take precaution against traveller’s diarrhoea [232].

### Prevention and Mitigation

Educating players and team staff is essential to mitigating the spread and impact of illness. Players must be aware of local pathogens they may encounter in host cities to allow informed choices about food and fluid consumption (e.g. drinking bottled versus tap water) [21, 233, 234]. For any diarrhoea-related issues, proper hydration and electrolyte replacement will help counteract diarrhoea-related dehydration [207] and reduce post-illness susceptibility to EHI/EHS [228]. For mosquito-borne and/or tick bite-related diseases, utilising insect repellents, wearing protective clothing and eliminating mosquito breeding sites (e.g. standing water) are effective [229, 230] alongside tick repellents, conducting regular tick checks and promptly removing ticks [235].

It is advisable for teams to remain informed about health advice from the local authorities and stay updated on changes to local health alerts. Furthermore, vaccination may be advisable to minimise the risk of infection during the tournament (aligned with World Health Organisation/local health authority guidelines) [21, 208]. Before proceeding with vaccination, teams must balance the risk–benefit of vaccinating players, whose needs differ significantly from the general population, ensure optimal timing (i.e. minimise impact of side effects on training/competition participation) and adopt appropriate strategies to reduce side effects (e.g. paracetamol to abate pain/fever) [236].

## Conclusions

The Men’s 2026 Football FWC will expose players to environmental conditions that will pose significant challenges to their health and performance: (i) extreme heat; (ii) altitude; (iii) air pollution and seasonal allergens; and (iv) travel. Previous FWC’s have not seen such a combination of extreme environmental factors for teams to mitigate. Despite a plethora of evidence-informed strategies to prepare for/mitigate against these environmental challenges, football-specific data are generally lacking and when present, suffer from limited external/ecological validity. This review uses the best available evidence to present guidelines to mitigate these challenges at the 2026 FWC. The teams that best adopt the outlined evidence-based guidelines into their practice will be best equipped to mitigate the impact of the environmental challenges at the 2026 FWC. A synthesis of these guidelines, optimising their integration into practice-compatible strategies, is presented in a partner review [40].

## Supplementary Information

Below is the link to the electronic supplementary material.

Supplementary file1 (PDF 24 KB)

## Abbreviations

CWC: Club World Cup
CWI: Cold-water immersion
Tc: Core temperature
EHI: Exertional heat illness
EHS: Exertional heat stroke
FIFA: Fédération Internationale de Football Association
FWC: FIFA World Cup
GMT: Greenwich mean time
HBmass: Haemoglobin mass
HR: Heart rate
HA: Heat acclimation/acclimatisation
HWI: Hot water immersion
LHTH: Live high-train high
LHTL: Live high-train low
LHTL + H: Live high-train low and high
LLTH: Live low-train high
LTHA: Long-term heat acclimation/acclimatisation
LA: Los Angeles
mmHg: Millimetre of mercury
NBA: National Basketball Association
NSAIDS: Non-steroidal anti-inflammatory drugs
O_2_: Oxygen
O_3_: Ozone
*p*O_2_: Partial pressure of oxygen
PM: Particulate matter
PHA: Passive heat acclimation
PV: Plasma volume
RSA: Repeated-sprint ability
RSH: Repeated-sprint training in hypoxia
STHA: Short-term heat acclimation/acclimatisation
Tsk: Skin temperature
USA: United States of America
WBGT: Wet-bulb globe temperature

## Declarations

### Data Availability Statement

No datasets were generated or analysed for the production of this article.
