# Supplementary material for: The 2026 Men’s FIFA Football World Cup: Evidence-Based Guidelines to Protect Player Health and Performance from Environmental Challenges
Source: Sports Med. 2026 Mar 24;56(6):1337–61. doi: 10.1007/s40279-026-02398-4 (PMC13260243; doi:10.1007/s40279-026-02398-4)
Supplement: Supplementary file 1 — Supplementary file1 (PDF 24 KB) [file 40279_2026_2398_MOESM1_ESM.pdf]

Supplementary material

Title: The 2026 Men's FIFA Football World Cup: Evidence-based guidelines to protect player health and performance from environmental challenges

Running heading: Environmental challenges at the 2026 Men's FIFA Football World Cup: Protecting player health and performance

Journal: Sports Medicine

Authors: Chris J Esh[1,2], Sarah Carter[3], Valérie Bougault[4, 5], Olivier Girard[6], Dina C Janse van Rensburg[7,8], Bryna C R Chrismas[2], Tim Meyer[9, 10], Lee Taylor[2,10,11]

Corresponding author: Dr Lee Taylor, School of Sport, Exercise and Health Sciences, Loughborough University, Epinal Way, Loughborough, LE11 3TU UK, (+44) 1509 226388, l.taylor2@lboro.ac.uk.

Supplementary Material

Methodology of air quality analysis

The daily Air Quality Index (AQI) values are reported for each city, and calculated according to the United States Environmental Protection Agency (US EPA). Data were collected from June 1 to July 31 for the years 2019 to 2024 and then averaged (366 days).

For U.S. cities, AQI data was obtained from the official EPA website [1]. The geographical area selected was the city itself, or if unavailable, the corresponding county.

For Mexican cities, some data was missing from the official government website [2], and additional data was obtained from AirNow [3] or Aqicn [4]. The AirNow interactive map or government website was used to identify the monitoring stations closest to the stadiums. For Mexico City, the worst AQI value among four surrounding stations that were relatively far from the stadium (stations 800090072, 800090073, 800090034, and 800090101), using Airnow was reported. AQI data for 2024 were not available for Mexico City, so the June and July data for the years 2019 to 2023 were averaged instead. For Guadalajara and Monterrey, AQI was not available on Airnow and official data was not fully available on the official website [2] at the time of the data search. Historical data for the Santa Margarita station, near the Guadalajara stadium, was unavailable. Therefore, the Aqicn platform was used to identify the closest available monitoring station to the stadium, even if it was ~10 km away. The stations chosen were Las Águilas for Guadalajara and Pastora for Monterrey. For Guadalajara, values from June and July of the previous years (2017 and 2018) were included to increase the amount of data compared to other cities (approximately 366 days for the other cities, and 292 days for Guadalajara). Days in each AQI category were only counted where both PM2.5 and ozone were measured. For Monterrey, data were analyzed from 2020 to 2024, as before 2020, only PM2.5 data were available for this station, and no ozone data. Finally, 245 days were included.

For Canadian cities, official regional websites: [5] (Vancouver) and [6] (Toronto) were used. The stations closest to the stadiums were Clark Drive for Vancouver and Toronto Downtown for Toronto. Using hourly pollutant concentration data the daily averages were calculated for PM2.5 and 8-hour averages for ozone. AQI values were then computed following the US EPA methodology [7].

Supplementary Table 1: Mean number of days per month (June and July) across US Air Quality Index categories (AQI)

	Number of days in each Air quality Index category [min-max]					
	**Green**	**Moderate**	**Unhealthy for sensitive individuals**	**Unhealthy**	**Very unhealthy**	**Hazardous**
**Western Region**						
Los Angeles	1 [0-4]	11 [0-24]	11 [2-18]	7 [1-18]	1 [0-4]	0 [0-0]
San Francisco	23 [17-29]	7 [2-14]	0 [0-1]	0 [0-1]	0 [0-0]	0 [0-0]
Seattle	22 [13-28]	7 [1-14]	1 [0-5]	1 [0-2]	0 [0-0]	0 [0-0]
Vancouver	29 [25-30]	2 [0-6]	0 [0-0]	0 [0-0]	0 [0-0]	0 [0-0]
**Central Region**						
Dallas	7 [2-17]	20 [8-27]	2 [0-6]	0 [0-1]	0 [0-1]	0 [0-0]
Guadalajara*	8 |1-18]	19 [12-28]	1 [0-4]	0 [0-0]	0 [0-0]	0 [0-0]
Houston	3 [0-9]	23 [17-29]	3 [0-7]	1[0-3]	0[0-1]	0 [0-0]
Kansas City	6 [0-15]	21 [13-28]	3 [0-12]	0 [0-1]	0 [0-0]	0 [0-0]
Mexico City	5 [0-13]	12 [8-20]	9 [5-12]	3 [1-9]	0 [0-3]	0 [0-0]
Monterrey	14 [4-22]	15 [7-22]	1 [1-1]	0 [0-0]	0 [0-0]	0 [0-0]
**Eastern Region**						
Atlanta	6 [0-11]	23 [16-26]	3 [0-7]	0 [0-1]	0 [0-0]	0 [0-0]
Boston	12 [6-19]	17 [11-24]	1 [0-4]	0 [0-1]	0 [0-0]	0 [0-0]
Miami	9 [2-19]	19 [2-26]	1 [0-4]	0 [0-0]	0 [0-1]	0 [0-0]
New York	16 [10-23]	13 [7-20]	1 [0-4]	0 [0-3]	0 [0-1]	0 [0-0]
Philadelphia	10 [3-19]	20 [14-30]	2 [1-5]	0 [0-3]	0 [0-1]	0 [0-1]
Toronto	17 [11-22]	14 [10-19]	0 [0-1]	0 [0-2]	0 [0-0]	0 [0-0]

Data expressed as mean per month [min-max]. Only months with more than 25 days measurements were noted. For Mexico City, data from 2024 is not included. *For Guadalajara included data were from June and July from years 2017 to 2019, July 2020, and June 2021 and 2024. For Monterrey, included data were from June to July 2020 to 2022 and July 2023.

References

1. USEPA. United States Environmental Protection Agency: Air quality index daily values report. 2025 [cited 2025 12/06]; Available from: https://www.epa.gov/outdoor-air-quality-data/air-quality-index-daily-values-report
2. SINAICA. Sistema Nacional de Información de la Calidad del Aire. 2025 [cited 2025 12/06]; Available from: https://sinaica.inecc.gob.mx/
3. AirNow. AirNow Air Quality. 2025 [cited 2025 12/06]; Available from: https://www.airnow.gov/
4. Aqicn. Air pollution in World. 2025 [cited 2025 12/06]; Available from: https://aqicn.org/map/world
5. Environment BCMo. BC Air Data Archive Website. 2025 [cited 2025 12/06]; Available from: https://envistaweb.env.gov.bc.ca/
6. Ontario AQ. Air Quality Health Index (AQHI) 2025 [cited 2025 12/06]; Available from: https://www.airqualityontario.com/history/index.php
7. AirNow. Technical assistance document for reporting the daily AQI. 2025 [cited 2025 12/06]; Available from: https://www.airnow.gov/publications/air-quality-index/technical-assistance-document-for-reporting-the-daily-aqi/
